# Single coronary artery originating from the ascending aorta

**DOI:** 10.1093/ehjcr/ytae210

**Published:** 2024-04-18

**Authors:** Pablo Vadillo Martín, Juan Francisco Cueva Recalde, David Ibañez Muñoz, Antonela Lukic Otanovic

**Affiliations:** A Cardiology Department, Hospital Clínico Universitario Lozano Blesa, Calle de San Juan Bosco, 15, 50009 Saragossa, Spain; A Cardiology Department, Hospital Clínico Universitario Lozano Blesa, Calle de San Juan Bosco, 15, 50009 Saragossa, Spain; A Radiology Department, Hospital Clínico Universitario Lozano Blesa, Saragossa, Spain; A Cardiology Department, Hospital Clínico Universitario Lozano Blesa, Calle de San Juan Bosco, 15, 50009 Saragossa, Spain

Coronary artery anomalies present major diagnostic and therapeutic challenges.^1^ Given the considerable heterogeneity of the coronary vasculature, the description of rare entities is essential to facilitate their cannulation in emergency situations. We present the uncommon case of a patient with a barely described coronary anomaly.

A 68-year-old male, asymptomatic to date, with cardiovascular risk factors (hypertension, diabetes, and dyslipidaemia) and family history of sudden aortic-related death in his father, presented in the emergency department with angina and ST-segment elevation in the inferior leads (*Figure 1A*).

**Figure 1:**
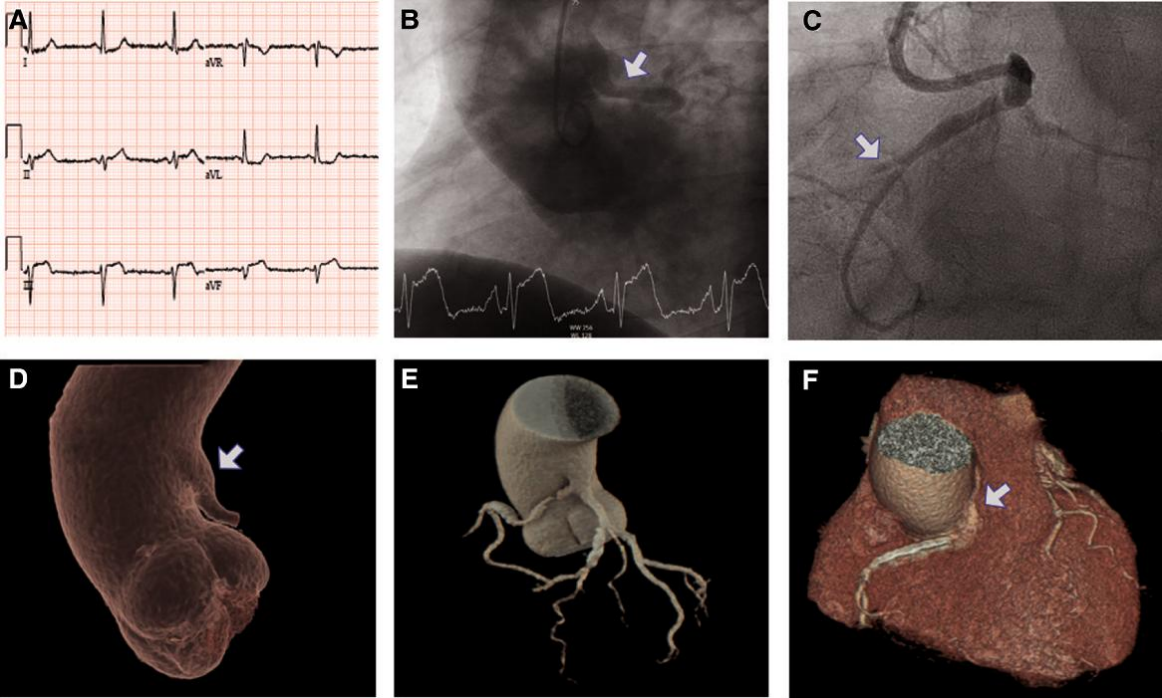
(*A*) Initial electrocardiogram: ST-segment elevation in the inferior leads. (*B*) Aortography: the arrow indicates the onset of single coronary artery originating from the ascending aorta. (*C*) Coronary angiography: the arrow indicates acute occlusion of the proximal–mid segment of the right coronary artery. (*D*, *E*) Computed tomography with 3D reconstruction: the arrow indicates the onset of single coronary artery originating from the ascending aorta. (*F*) Aorto-coronary computed tomography: the arrow depicts interarterial course of the right coronary artery between the aorta and the pulmonary artery.

After a long and complex coronary artery cannulation, emergent coronary angiography showed the presence of a single coronary artery with a high origin in the ascending aorta, with an acute occlusion of the proximal–mid segment of the right coronary artery (RCA; *Figure 1B* and *C*). After opening the vessel, the presence of a complicated atherosclerotic plaque was visualized, and percutaneous transluminal coronary angioplasty was performed with implantation of a drug-eluting stent, with satisfactory results.

Given the presence of ascending aorta dilation in the echocardiogram, an aorto-coronary computed tomography (CT) with cardiac synchronization and prospective acquisition was performed, which allowed us to describe the presence of interarterial course of the RCA between the aorta and the pulmonary artery (*Figure 1D–F*). The patient progressed favourably with no anginal recurrence during follow-up. Given his age and lack of previous symptoms in relation to his coronary anomaly, it was decided to perform conservative management.

The anomalous origin of the coronary arteries is rare, but its identification is crucial for the management of patients with associated coronary artery disease.^2,3^ These anatomical alterations can be lethal or lead to a worse outcome in a patient with an acute pathology as in our case.

**Consent:** Appropriate consent has been obtained from the patient for the completion and publication of the clinical case, confirming compliance with the COPE guidelines.

**Funding:** None declared.

## Data Availability

The data underlying this article will be shared upon reasonable request to the corresponding author.
